# Triple Vessel Disease With Intracranial Hemorrhage in a Patient With Guillain–Barre Syndrome: A Potential Complication, a Coincidental Finding or a Treatment‐Related Adverse Event

**DOI:** 10.1002/ccr3.71554

**Published:** 2025-11-29

**Authors:** Sagun Ghimire, Shikher Shrestha, Dinuj Shrestha, Ananta Maharjan, Kritick Bhandari, Suman Bhattarai, Kajan Ranabhat

**Affiliations:** ^1^ Department of Neurosurgery B and B Hospital Lalitpur Nepal; ^2^ KIST Medical College and Teaching Hospital Lalitpur Nepal; ^3^ Department of Neuroscience B and B Hospital Lalitpur Nepal; ^4^ Department of Radiology B and B Hospital Lalitpur Nepal

**Keywords:** Guillain–Barre syndrome, intracranial hemorrhage, neurosurgery, triple vessel disease

## Abstract

Triple vessel disease and intracranial hemorrhage complicating Guillain–Barre syndrome have been rarely reported. Furthermore, there are no established theories that explain the pathophysiological association between these three different clinical scenarios. Despite the treatment‐related quandary in this case, a good outcome was eventually achieved.

## 1 Introduction

Guillain–Barre syndrome (GBS), triple vessel disease (TVD), and intracranial hemorrhage (ICH) are three different clinical conditions resulting in grievous life‐threatening insults to the body. There is no pertinent literature that elaborates on the pathophysiological associations among these three separate disease conditions. While some evidence [1] suggests GBS secondary to ICH, in our case, ICH along with TVD followed GBS. The occurrence of ICH and TVD following GBS adds further complexity to treating patients who are already critically ill. Moreover, no treatment guidelines exist to curtail the use of intravenous immunoglobulin (IVIg) in cases where GBS is associated with other multiple systemic complications. In such cases of multisystem involvement, it becomes difficult to determine whether the subsequent complications are due to the idiopathic pathophysiological mechanisms of the disease, coincidental findings, or just mere treatment‐related adverse event, hence creating a diagnostic and treatment‐related dilemma.

## 2 Case History/Examination

A 54‐year‐old female with recently diagnosed systemic hypertension presented to the Department of Neurosciences of our hospital with the complaint of weakness of bilateral upper and lower limbs since 1 week and increasing severity since the last 3 days. The weakness was acute in onset, which started on its own without any significant preceding history aside from recent travel from another country. During her presentation to the outpatient department, the patient was unable to ambulate independently and had been wheelchair bound since Day 1. According to the patient, the weakness started in the bilateral lower limbs and slowly progressed to upper limbs, and was associated with severe burning and tingling sensations along with numbness and lower back pain. There was no history of systemic symptoms such as fever, loss of consciousness, abnormal body movements, difficulty in breathing, shortness of breath, features of acute gastroenteritis, bowel and bladder incontinence, chest pain, or palpitations, but the patient complained of increased urinary frequency since 3 days without other symptoms of urinary tract infections. On systemic examination, all vital signs were within normal limits. Neurological examination revealed motor power of Medical Research Council (MRC) grade 4/5 in the bilateral upper limbs and 3/5 in the bilateral lower limbs. Reflexes were absent in the bilateral upper biceps, triceps, and bilateral lower knees and ankles. The plantar reflex was mute bilaterally. On sensory examination, sensation was intact over bilateral sensory dermatomes but the patient reported burning and tingling sensations. Cranial nerve examination showed all cranial nerves to be grossly intact. Examination for suspected lumbar prolapsed intervertebral disc (PIVD) was performed. The straight leg raising test (SLRT) was limited to 20–30 degrees bilaterally due to weakness. Bilateral External Hallucis Longus (EHL) power was 3/5, and bilateral dorsiflexion/plantar flexion was 3/5.

## 3 Methods (Differential Diagnosis, Investigations, and Treatment)

**FIGURE 1:**
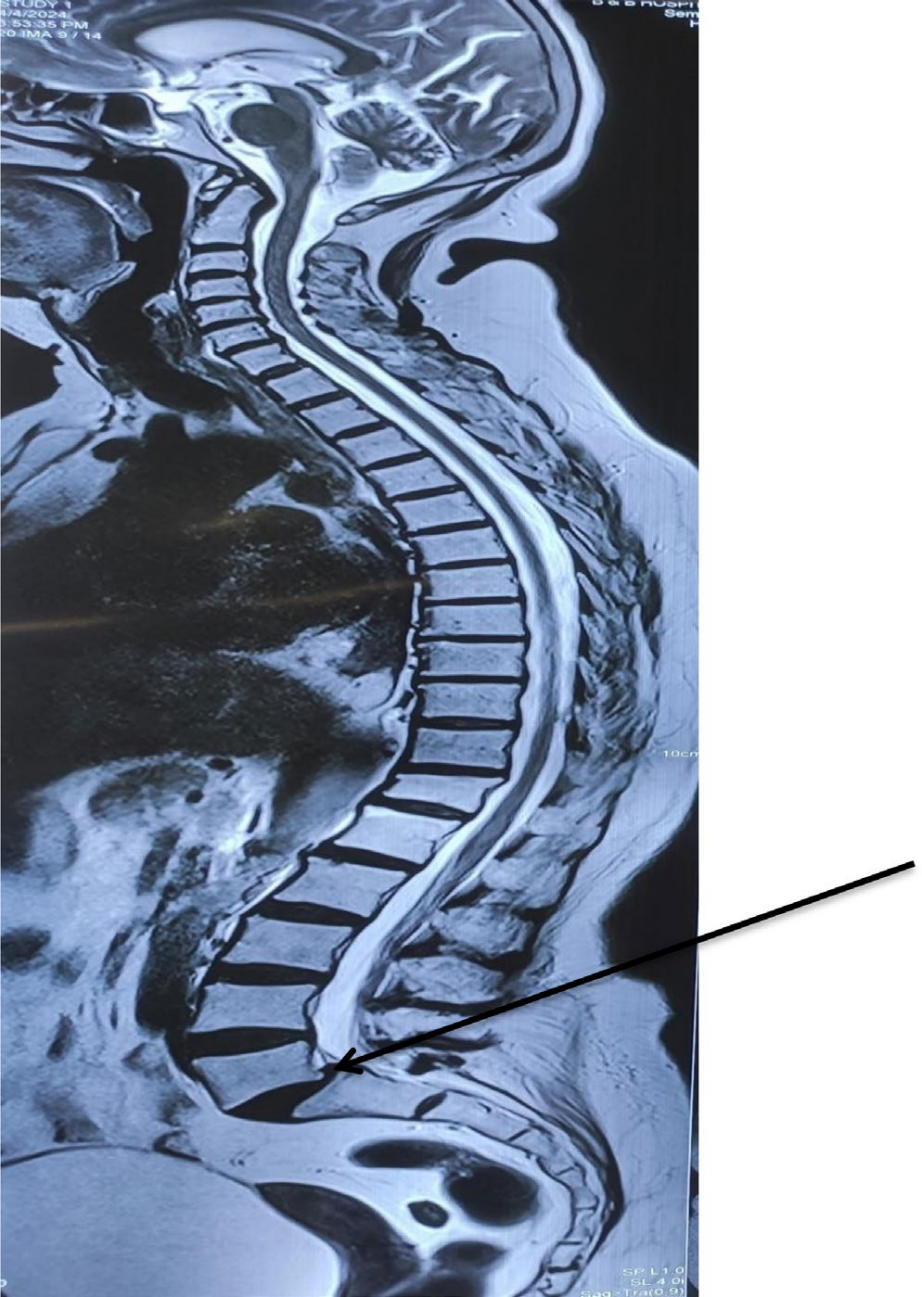
MRI whole spine screening shows features suggestive of posterior subluxation of L5‐S1 joint (black arrow) without any obvious spinal cord or nerve root compression.

**FIGURE 2:**
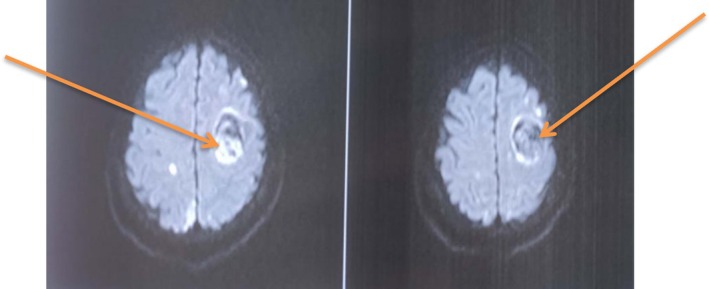
MRI brain DWI sequence shows left‐sided intracranial hemorrhage (orange arrows).

**FIGURE 3:**
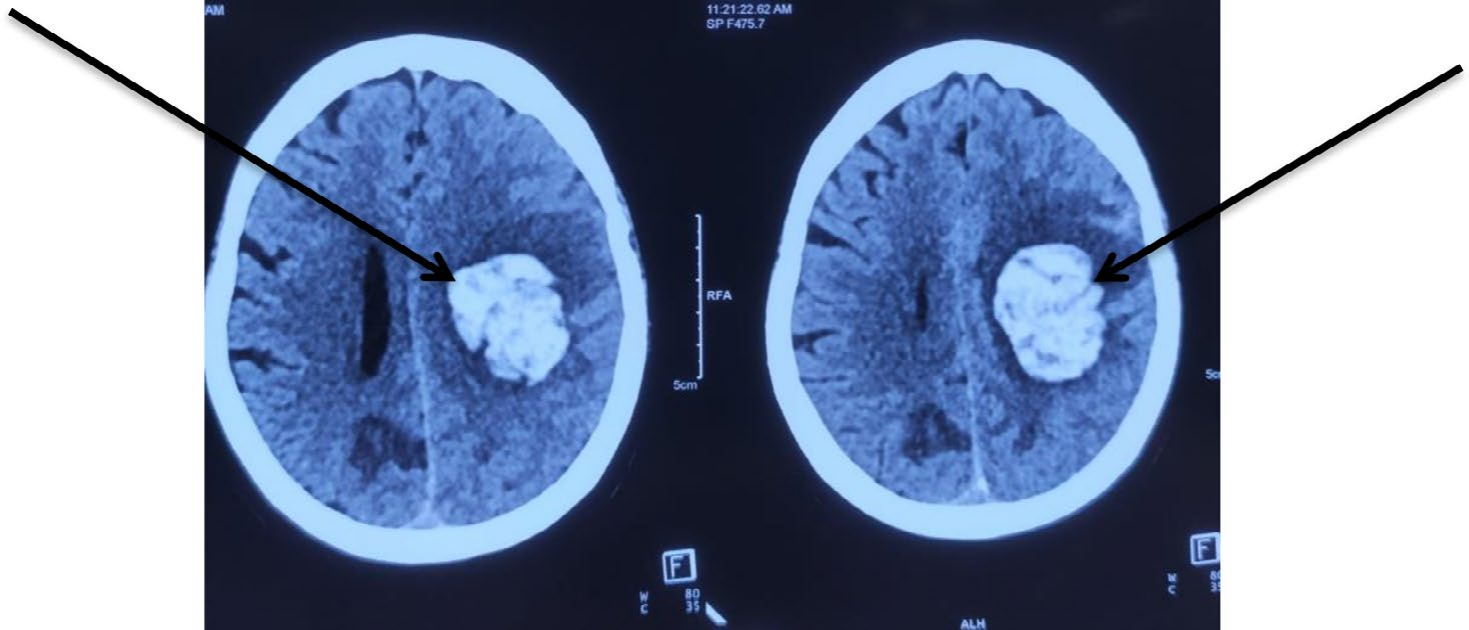
Plain CT head showing hyperdense lesion over left fronto‐parietal region suggestive of ICH (black arrows) measuring around 30 mL.

**FIGURE 4:**
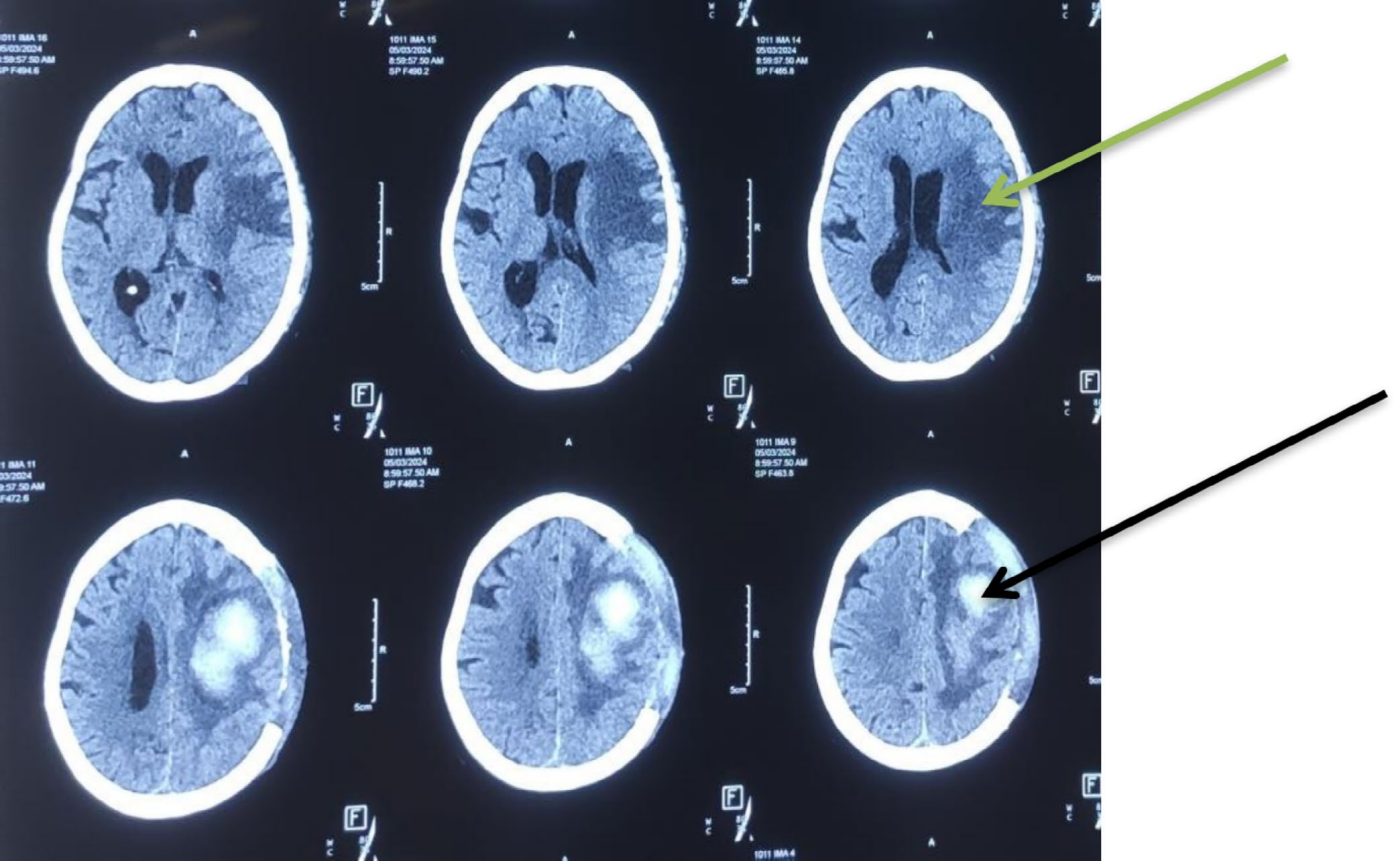
Post‐left fronto‐temporo‐parietal decompressive craniectomy plain CT head showing craniectomy changes of left side with cerebral edema (green arrow) surrounding the ICH core (black arrow) without any obvious midline shift.

In view of lower back pain and acute onset of four limb weakness, the patient initially underwent an MRI of the lumbo‐sacral spine to rule out suspected cauda equina syndrome. However, the MRI of the lumbo‐sacral spine showed only posterior subluxation of the L5‐S1 joint with no obvious spinal cord or nerve root compression (Figure 1). Her complete blood count, renal function test, liver function test, and PT/INR were all within normal limits, but her urine routine examination showed 8–10 pus cells and hence empirical antibiotics were started. Over the subsequent days, the weakness further progressed in an ascending nature with motor power deteriorating to 2/5 in both bilateral lower and upper limbs. Given the suspicion of GBS, a nerve conduction study was performed which showed mixed axonal/demyelinating sensorimotor polyneuropathy with features of conduction block over the lower limbs (common peroneal nerve and posterior tibial nerve). These findings were consistent with an acute demyelinating polyneuropathy, specifically the acute motor sensory axonal neuropathy (AMSAN) variant. To further confirm the diagnosis of GBS, cerebrospinal fluid (CSF) analysis was also performed. It showed elevated CSF protein (> 0.55 g/L) without white blood cell count, consistent with albuminocytological dissociation. Hence, IVIg was initiated at the dose of 0.4 g/kg, administered as 20 g over 6 h for 5 days. Following her second cycle of IVIg, there was marked symptomatic improvement including decreased burning, tingling sensation along with improved power of bilateral upper and lower limbs. However, after completing her second cycle of IVIg, her EKG showed features suggestive of T wave inversion over leads II, III and aVF. Given her history of ischemic heart disease, anticoagulants were initiated: Tab aspirin (75 mg), Tab clopidogrel (75 mg), and Inj enoxaparin (40 mg, sc). To monitor coagulation, the patient's prothrombin time (PT), international normalized ratio (INR), activated partial thromboplastin time (APTT), bleeding time/clotting time (BT/CT), fibrinogen degradation products (FDP), and factor Xa were checked every alternate days. All results were within normal limits. A coronary angiogram (CAG) was also carried out, which showed 95% stenosis in the proximal and 75% stenosis in the distal right coronary artery, complete total occlusion in the posterior descending artery (PDA), 75% stenosis in proximal to mid left anterior descending (LAD), 75% stenosis in mid left circumflex (LCX). This led to a diagnosis of TVD and percutaneous intervention (PCI) was planned once the patient stabilized. Furthermore, IVIg was continued. However, after completing her 5th cycle, the patient complained of isolated acute and abrupt right‐sided hemiparesis suggestive of stroke‐like features. A brain MRI (Figure 2) was performed which showed left‐sided ICH measuring around 10 mL in volume. Hence, conservative management was planned and all the anticoagulants were stopped. In subsequent days, the patient's drowsiness worsened, accompanied by persistent severe weakness. A repeat NCCT head was carried out which showed expansion of ICH measuring to 30–35 mL (Figure 3). Therefore, the patient was scheduled for decompressive craniectomy and evacuation of ICH (Figure 4).

## 4 Conclusions and Results (Outcome and Follow Up)

Post‐operatively, the patient required mechanical ventilation for 2 months. Due to the prolonged need for ventilator support, the patient underwent tracheostomy on post‐operative day 15. Throughout her ICU stay, her GCS indicated spontaneous unpurposeful eye opening and localization to pain. After more than 2 months in ICU on mechanical ventilation, she was gradually weaned off to T‐piece and finally extubated on her post‐operative day 64. The patient was later discharged from ICU to her home under the continued use of a metallic tracheostomy tube and NG feeding. Her home care regimen included home nursing care, and chest and limb physiotherapy. On her second month follow‐up visit, her GCS showed spontaneous purposeful eye opening and she obeyed command. She continued with a metallic tracheostomy tube and NG feeding, along with regular physiotherapy. No other systemic complications were noted and the patient was hemodynamically stable.

## 5 Discussion

This case report describes a patient with GBS presenting with myocardial infarction, TVD, and ICH. GBS is a rare but serious post‐infectious immune‐mediated neuropathy resulting from the autoimmune destruction of nerves in the peripheral nervous system. It causes symptoms such as numbness, tingling, and weakness that can progress to paralysis [2]. The diagnosis of GBS is mostly clinical. Electromyography and nerve conduction studies may be helpful in distinguishing GBS from its differential diagnosis. CSF analysis shows a classic pattern of albuminocytologic dissociation [3]. The most feared complications are respiratory compromise and bulbar palsies; however, they can rarely be associated with cardiovascular complications as well. An observational study revealed that 54.2% of GBS patients experienced cardiovascular complications. Among these, electrocardiography (ECG) findings (50%), hypertension (28.12%), labile hypertension (12.5%), tachycardia (26.04%), bradycardia (13.54%), and a fluctuating heart rate (HR, 11.46%) were the most prominent findings but either dual vessel or triple vessel disease was not found to be associated [4]. The pathophysiology of cardiovascular complications in GBS is complex. These complications are observed in around two‐thirds of affected patients and are mostly attributable to autonomic dysfunction, which is a major threat to patient survival but it was not observed in our study [5]. These complications include, but are not limited to, heart rate variability, hypertension or hypotension, cardiomyopathy, acute coronary syndrome, and electrocardiographic changes [6]. Catecholamine‐associated myocardial injury theories can partly explain such complications suggesting the role of a disturbance of catecholamine uptake surrounding myocytes, redistribution of coronary blood flow, and myocardium denervation hypersensitivity [7].

Any GBS patient with chest pain and abnormal ECG should be considered myocardial infarction until proven otherwise. There also arises a need to undergo cardioangiogram as well to determine whether there is a need for percutaneous intervention or not. The sensory manifestation of GBS with burning sensation over the limbs and occasionally over the chest may obscure the diagnosis of MI as the suspicion gets misdirected towards atypical angina or any other treatment‐related adverse events in cases of critical patients [7]. Our patient initially only had bilateral lower limb weakness and no sensory weakness. However, after completing two cycles of IVIg, the ECG showed an abnormal feature with T wave inversion in II, III, and aVF. Subsequently, a CAG revealed TVD. Unlike other cases of MI, our patient did not have the classical atherosclerotic risk factors such as smoking, obesity, dyslipidemia, diabetes mellitus, chronic hypertension, renal dysfunction, and prior coronary or cerebrovascular disease. This occurrence has been reported in several case reports where IVIg administration resulted in coronary occlusion in GBS patients, attributed to the increased prothrombotic risk subsequent to immunoglobulin administration [8, 9, 10]. However, the concurrent association of TVD and ICH observed in our case was difficult to explain based on existing medical literature that describes pertinent pathophysiological mechanisms. Thrombotic events are reported in most clinical conditions where IVIg has been used, with an estimated incidence of 0.15%–1.2% per treatment course [10]. Platelet activation, increased blood viscosity, contamination of IVIg with activated coagulation factors, induced arterial vasospasm, production of vasoconstrictive cytokines, and vasculitis are some of the proposed mechanisms of thrombosis due to IVIg [11]. Our patient also experienced intracerebral hemorrhage and whether this was a coincidental occurrence or an entirely new pathophysiologic relation can be debatable. Knowledge regarding this is limited to case reports, and it has been postulated that CNS hemorrhage following GBS can be due to blood vessel autonomic dysfunction and the intravenous use of immunoglobulin [12]. To reduce IVIg‐related thrombosis, recommendations include maintenance of hydration, a slower rate of infusion, combined with antiplatelet therapy and anticoagulation [13, 14, 15, 16]. However, in cases of multiple systemic involvement, such as in our case, it is difficult to establish a definitive treatment protocol. Hence, further investigation into the underlying mechanisms of such complex associations is needed to improve the outcome in cases of critically ill patients.

## Author Contributions

**Sagun Ghimire:** conceptualization, methodology, project administration. **Shikher Shrestha:** conceptualization, investigation, supervision. **Dinuj Shrestha:** supervision, visualization. **Ananta Maharjan:** resources, supervision, visualization. **Suman Bhattarai:** investigation, methodology. **Kritick Bhandari:** resources, writing – original draft. **Kajan Ranabhat:** investigation, supervision.

## Funding

The authors have nothing to report.

## Disclosure

Consent to Publish: The participant has consented to the submission of the case report to the journal.

## Consent

Written informed consent was obtained from the patient to publish this report in accordance with the journal's patient consent policy.

## Conflicts of Interest

The authors declare no conflicts of interest.

## Data Availability

Any data used in the manuscript can be made available if asked by the chief editor.
